# What do we know about dietary perceptions and beliefs of patients with rheumatoid arthritis? A scoping review

**DOI:** 10.1007/s00296-024-05691-5

**Published:** 2024-08-27

**Authors:** Mario Termine, Zoe Davidson, Tammie Choi, Michelle Leech

**Affiliations:** 1 Faculty of Medicine Nursing and Health Sciences, Monash University, 27 Rainforest Walk, Clayton, VIC 3168 Australia; 2 Department of Nutrition, Dietetics and Food, School of Clinical Sciences, Monash University, 264 Ferntree Gully Road, Notting Hill, VIC 3168 Australia

**Keywords:** Rheumatoid arthritis, Diet, Beliefs, Health behaviours, Disease activity

## Abstract

Rheumatoid arthritis is a debilitating inflammatory condition which has a high disease burden. While there is emerging evidence that certain foods and diets could have anti-inflammatory properties and there are published ‘anti-inflammatory’ diets, there is very little understanding of patient beliefs and perceptions about the impact of diet on symptom management or attitudes to particular dietary interventions. This scoping review aims to summarize the existing literature around the beliefs that patients with rheumatoid arthritis hold regarding the impact of diet on disease activity and joint pain. It also examines the current state of evidence regarding the impact of specific dietary interventions on patient reported and objective parameters of RA disease activity. A search was conducted across seven databases for studies which included reporting on dietary beliefs related to disease management or investigations on the effect of particular diets on disease activity or joint pain. Articles were excluded if they examined extracted compounds or individual dietary supplements. Included studies were synthesized narratively. We retrieved 25,585 papers from which 68 were included in this review: 7 assessed dietary beliefs, 61 explored dietary interventions. The available literature on patient beliefs has been largely limited to quantitative studies with limited qualitative exploration. The Mediterranean, fasting and vegan diets appear to have the most benefit with regards to rheumatoid arthritis outcomes for patients. Research which examines RA patient’s beliefs and attitudes about the impact of diet on their RA symptoms and disease is currently lacking.

## Introduction

Rheumatoid Arthritis (RA) is the most common autoimmune joint disease and interventions rely on disease management through medications, physical therapy, or joint replacement surgery [1]. Many current medications which are considered standard of care for RA have side effects, such as an increased risk of infection, which reduce patient compliance [2]. There has been much interest in the impact of the environment and diet to modify and prevent RA disease in genetically at risk probands and mitigate the need for immunosuppressive therapies [3]. It is well known that diet plays a crucial part in chronic disease management and diet has become a major topic of interest for inflammatory diseases, especially RA [4]. It is common for patients to ask what they should eat, and dietary advice fact sheets are often provided by peak bodies [5]. These are mostly based on heart health and anti-inflammatory concepts, but are not specific for RA. Recent international randomised control trials have begun to explore the impact of specific diets such as fasting, Mediterranean, and vegan, on RA symptoms [6]. Furthermore, a recently published systematic review investigated dietary interventions in RA and demonstrated a positive link between diets such as Fasting and the Mediterranean diet, and a reduction in RA activity [3]. Despite this, to date, there are no dietary guidelines specifically for the management of RA to assist patients to better manage RA symptoms and existing guidelines are more focussed on pharmacologic intervention [7]. Importantly the recently released Australian Rheumatoid arthritis clinical care guidelines emphasise the importance of encouraging self-management [8]. Firmer and more specific RA dietary guidelines are likely to emerge in the near future. In this context it is critical to examine whether patients themselves believe in, or would be prepared to attempt, dietary approaches.

While there appear to be many studies which demonstrate that anti-inflammatory diets such as the Mediterranean, vegan and vegetarian are able to reduce pain and symptoms in RA patients, dietary beliefs of patients with RA are unknown and have not been systematically explored [9]. There is a need to investigate and unpack patients’ dietary beliefs to identify potential barriers which inhibit following an anti-inflammatory diet. This will better assist in influencing food choices and promote sustainable behavioural changes [10]. There are several models of behavioural change, such as the Health Belief model, which acknowledges an individual’s perceived susceptibility and severity of disease, and perceived benefits and barriers of action [11]. The exploration of dietary beliefs and behaviours could inform dietary guidelines and lead to an understanding of what types of foods RA patients eat, why they eat them, and how we can improve on their dietary intake in a way which also increase the likelihood of patients adhering to feasible dietary choices for symptom management.

A preliminary search of the Cochrane Library, JBI Evidence Synthesis and Medline was conducted on the 5th of April 2021. As this was a preliminary search, only keywords ‘rheumatoid arthritis’ and ‘diet’ were included to identify whether there was a paucity of research in this topic area. This yielded 21 review articles across all listed databases relevant to the topic of RA and diet. However, there were no reviews which focused on the dietary beliefs of RA patients as a key concept. No other current or underway systematic or scoping reviews were identified. This preliminary search identified a gap in the literature where dietary beliefs are not yet adequately established in patients with RA. A scoping review was therefore conducted so that a full synthesis of existing research could give a broader understanding of existing knowledge regarding dietary beliefs in RA patients. The primary aim of this scoping review was to describe the literature that considers the dietary beliefs of patients with RA, identifying the common dietary beliefs of RA patients. The secondary aim was to identify and collate existing literature exploring the impact of diet on RA symptoms.

## Methods

This scoping review utilised JBI methodology for scoping reviews [12]. This included utilisation of methodology inclusion criteria, search strategy and data extraction. This scoping review is reported in accordance with the PRISMA-ScR reporting guidelines [13].

### Eligibility criteria

Identified articles were required to meet either the primary or secondary aims of this paper for inclusion. Papers were included for the primary aim if they involved RA patients and investigated patient dietary beliefs. Papers were included in the secondary aim if they investigated the effect of diet on RA symptoms such as DAS-28, pain, morning stiffness and swollen or tender joints. Articles were included in this review if they involved adult RA patients, English language, peer-reviewed (interventional and observational), and considered a whole of diet approach. Articles were excluded if they were conference abstracts, protocol papers, reviews, theses/ dissertations, case reports, involved patients with other complicating diseases or infections, solely evaluated the effects of extracted compounds or micronutrients, or focused on single foods or single meals. The rationale behind this eligibility criteria was to ensure that only articles which captured whole diets were included in the final review. There was no subcategorization according to geographic location, race, or gender. Search limits applied on the databases included humans and English language. There were no limitations on publication date.

### Search strategy

The search was conducted on 21st May 2021 utilising six article databases including CINHAL, Cochrane Library, Embase, OVID Medline, Scopus and Web of Science. These databases were chosen as they are known to have the largest repertoire of health and science articles and, combined, they should capture all relevant articles to the topic. A Patient, Intervention, Comparison and Outcomes (PICO) table was created to assist with the search strategy across the databases (\* MERGEFORMAT Table 1). Key search terms were adapted across all six databases and English language and human limits were applied. The search was tested using key papers identified via Google Scholar. An updated search utilising the same methodology, six article databases and key search terms as per Table 1 was completed on the 1st July 2023 to ensure all relevant articles up to and including the 30th June 2023 have been included in this scoping review. A final search was completed on the 30th July 2024 including databases DOAJ, OVID Medline and Scopus using the search terms ‘rheumatoid arthritis’ and ‘diet’ to ensure all relevant articles up to and including 30th June 2024 have been included in this review.

**Table 1:** PICO Table describing key terms used for databases CINAHL, Cochrane Library, Embase, Medline (OVID), Scopus and Web of Science

Population	Intervention	Outcome
1. Exp^a^: rheumatoid Arthritis/^b^ 2. Rheumat*^c^ 3. 1 or 2	4. Exp: diet/ 5. Exp: nutrition therapy/ or exp: nutrition/ 6. Diet* 7. Nutri* 8. Food* 9. Intake* 10. Consumption 11. Feed* 12. 4 or 5 or 6 or 7 or 8 or 9 or 10 or 11	13. Exp: arthralgia/ 14. Exp: joints/ 15. Joint pain 16. Joint* 17. Fatigue/ 18. Exhaust* 19. Belie* 20. Tire* 21. Perce* 22. Attitude* 23. Behav* 24. Feel* 25. Knowledge 26. Preference* 27. Disease Activity 28. Daily Living 29. Quality of Life 30. Pain* 31. Fatigue* 32. DAS28 33. PGA 34. Physician global assessment 35. CRP 36. Exp: C-reactive protein/ 37. C-reactive protein 38. ESR 39. Erythrocyte sedimentation rate 40. 13 or 14 or 15 or 16 or 17 or 18 or 19 or 20 or 21 or 22 or 22 or 23 or 24 or 25 or 26 or 27 or 28 or 29 or 30 or 31 or 32 or 33 or 34 or 35 or 36 or 37 or 38 or 39 41. 3 and 12 and 40 42. Limit 41 to English Language and humans

^a^Exp: Explode for terms mapped to subject headings

^b^Medical Subject Headings

^c,^*: Truncation

### Study selection

Articles retrieved in the search were exported from the databases into EndNote X9.3.3, Bld 13,966. They were then collated and uploaded into Covidence V2785, 2022. Duplicate citations were first identified and removed using Covidence. Articles were screened by title and abstract by two independent reviewers (MT, ML, ZD) who categorised them as either eligible or irrelevant. Both reviewers had to agree on the categorisation and any conflicts were resolved by the third reviewer. Full texts were then retrieved for eligible papers. Again, two independent reviewers screened the articles (MT, ML, ZD) and categorised the articles as included or excluded, also having to agree on the reason for exclusion and having the third reviewer resolve conflicts. The same process was utilised for the updated search where two independent reviewers (MT, ML, TC) screened and categorised articles with a third reviewer resolving any conflicts.

### Data extraction

Data extraction was performed on all studies that met the inclusion criteria with information being entered into a Microsoft Excel table. The articles were divided into two groups; those that answered the primary question of this review and those that answered the secondary question. Articles which addressed the primary question were those which included any information regarding patient dietary beliefs, and all other articles were filtered into the secondary question. Data to be extracted was piloted for five articles addressing the primary and secondary question, reviewed by MT, ML and ZD, and then adapted into a final version before being expanded to the remaining articles. A detailed data extraction was performed by MT on the articles addressing the primary question with information collected on author, year, study design, location, sample size, sex, age, study groups, dietary outcomes, RA outcomes, inclusion criteria, exclusion criteria, how dietary beliefs were assessed, dietary questions, disease activity assessment, study duration, findings, and design weaknesses. An abbreviated data extraction was conducted on the studies which addressed the secondary question with information collected on author, year, study design, location, sample size, aims, dietary intake, RA outcomes and findings.

### Data synthesis

Data was synthesised in table format according to the review questions. For the primary review aim, a detailed data summary was provided with results from individual studies presented. For the secondary review question, a rudimentary synthesis was conducted to illustrate what diets or dietary patterns have been investigated in the literature and the effect of these on RA outcomes. A further breakdown of the articles addressing the second question was conducted and split into whether they focussed on a common and defined diet i.e. vegan or Mediterranean, for example, or whether there was another observed dietary pattern.

## Results

**Fig. 1:**
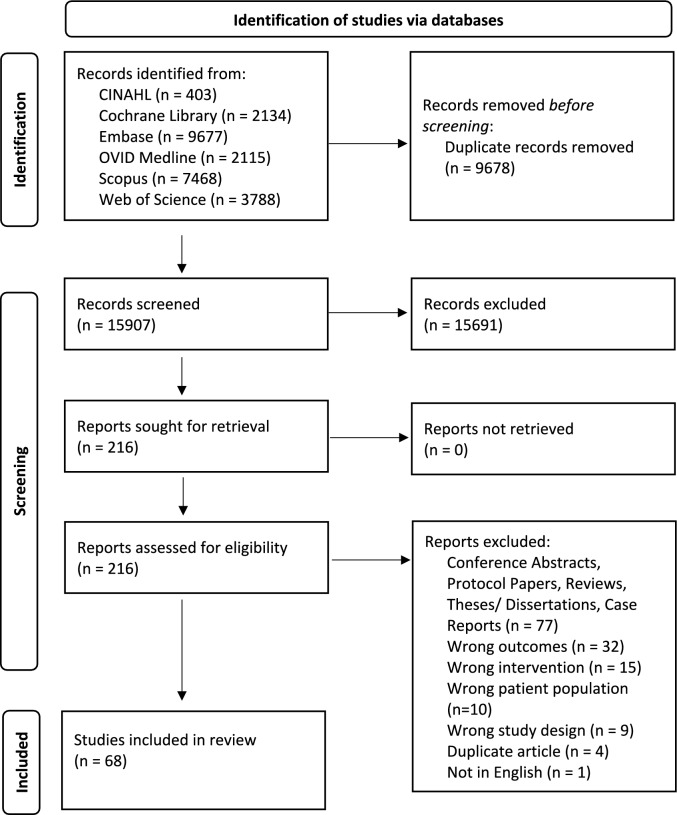
PRISMA flow diagram of articles identified through CINAHL, Cochrane Library, Embase, OVID Medline, Scopus and Web of Science [75]

**Fig. 2:**
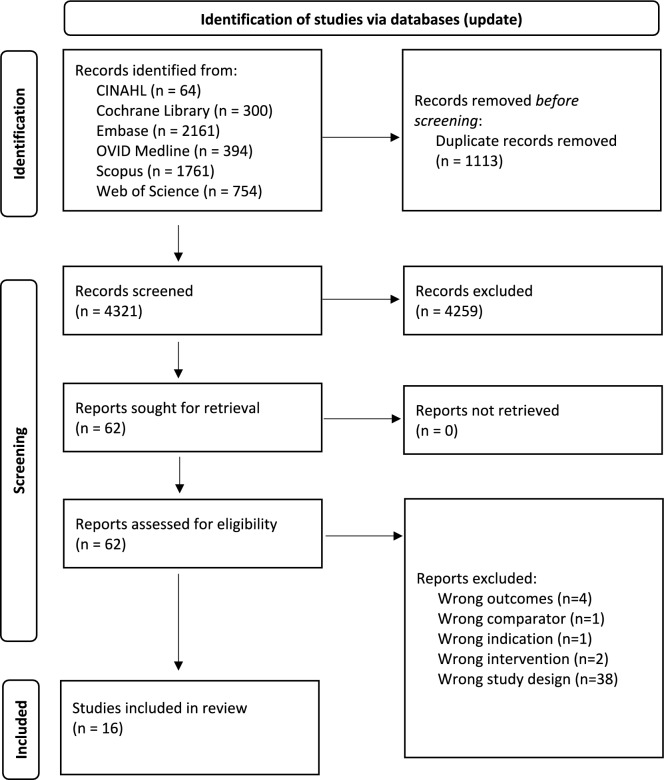
PRISMA flow diagram of articles identified through updated search of CINAHL, Cochrane Library, Embase, OVID Medline, Scopus and Web of Science [75]

From this initial search, there were 25,585 articles identified across all databases. Covidence identified 9678 duplicates which were removed for the next step, leaving 15,907 unique articles to be screened. After screening by title and abstract, and then by full article, there were 68 articles included in the final analysis for this scoping review (Fig. 1). From these, seven articles addressed the primary question and 61 addressed the secondary question. Of those identified for the secondary question, 42 articles explored common diets and 11 observed other dietary patterns. The results of the literature search have been divided into three sections each of which focus on the aforementioned aspects. The updated search yielded a further 5434 of which 1113 were identified as duplicates. After screening by title and abstract, and then by full article, there were 16 additional articles included for final analysis, one of which addressed the primary question and 15 which addressed the secondary question (\* MERGEFORMAT Fig. 2). The final search yielded 401 articles, of which one was found to be relevant to the primary question and four to be relevant to the secondary question.

### Overall review of dietary beliefs and attitudes

Only eight studies were identified that explored dietary beliefs of patients with RA (\* MERGEFORMAT Table 2). The average age of participants in the studies was 55 years old, ranging from 39 to 65; and the majority of participants were women. The sample sizes varied from 15 to 432 participants and data collection was focused on patient questionnaires. These studies were conducted in United Kingdom [14], France [15], Poland [16], Norway [17], India [18], Finland [19], United States of America [20, 21] and Ireland [22]. Across the countries, they were conducted intermittently and quite sporadically from 1991 to the latest publication in 2023.

**Table 2:** Dietary beliefs of patients with rheumatoid arthritis and effect on disease activity

Study	Country	Design	Sample size (n) and demographics	Believers (b) and non-believers (d)	Data collection method	Dietary attitude/belief	RA Activity Outcomes
Goff et al., 1999 [14]	United Kingdom	Observational	n = 23 Average Age = 57 years Average Disease Duration = 20.5 years	b = 13 (56%) d = 10 (44%)	Patient questionnaire	Citrus fruits, tomatoes, vinegar, pickles, dairy products, red meat and alcohol were believed to negatively affect symptoms	Better self-reported mobility was found in patients who believed diet to impact disease outcomes
Gossec et al., 2018 [15]	France	Cross-sectional, prospective	n = 432 Average Age = 58.3 years Women = 276 Average Disease duration = 13.1 years	b = 45 (12%) d = 387 (88%)	Patient questionnaire	Eating certain foods can trigger or reduce rheumatoid arthritis flares	RA outcomes were not analysed in the context of dietary beliefs
Grygielska et al., 2017 [16]	Poland	Observational	n = 165 Average Age = 56.5 years Women = 87%	b = 101 (61%) d = 64 (39%)	Patient questionnaire	Diet affects health of rheumatic disease	Comparison of RA activity outcome between believers and non-believers were not evaluated in this study
Haugen et al., 1991 [17]	Norway	Observational	n = 184 Median Age = 58 years Median disease duration = 11 years	b = 69 (38%) d = 115 (62%)	Patient questionnaire	Diet has a great influence on disease symptoms in rheumatic diseases	Disease outcomes of RA activity were not reported
Munshi et al., 2008 [18]	India	Observational	n = 15 Average Age = 39 years Females = 15 Average disease duration = 53 months	b = 9 (60%) d = 6 (40%)	Patient questionnaire	Diet has a great influence on disease symptoms in rheumatic diseases	Disease outcomes of RA activity were not reported
Salminen et al., 2002 [19]	Finland	Observational	n = 104	b = 42 (40%) d = 62 (60%)	Patient questionnaire	There is a connection between diet and rheumatoid arthritis disease	Disease outcomes of RA activity were not reported
Tedeschi et al., 2017 [20]	United States of America	Observational	n = 217 Average age = 65 years Female = 180 Average disease duration = 17 years	b = 52 (24%) d = 165 (76%)	Patient questionnaire	Specific foods such as blueberries, fish, strawberries, spinach, tomato, diet soda, eggplant, red meat, beer, desserts, soda with sugar have an effect on RA symptoms	No significant differences in DAS28-CRP, CDAI, M-HAQ and RADAI between believers and non-believers
McGarrity-Yoder et al., 2022 [21]	Arizona, U.S.A	Observational	n = 50 Average age = 60 years Females = 34	b = 22 (44%) d = 28 (56%)	Food frequency questionnaire	Investigate associations between diet quality and rheumatoid arthritis disease activity	No significant differences in DAS28, HAQ, VAS pain, ESR or CRP between believers and non-believers
Raad et al,. 2023 [22]	Limerick, Ireland	Interventional	n = 21 Average age = 47.5 years Females = 20	Not reported	Patient interviews	Heating a healthy diet will help patients feel better	Disease outcomes not reported in this study

The exploration of dietary beliefs in most of the identified studies involved asking patients questions such as ‘How great an influence in general is diet believed to have on disease symptoms?’ and ‘What diet are you currently on?’. Only one study asked patients to specify whether they avoided any foods, cooked foods in a special way or ate their meals at a certain time [14]. Of the nine studies, three reported a higher proportion of patients believing that diet influenced their RA [14, 16, 18] while five studies reported more patients who did not believe their diet significantly impacted their RA [15, 17, 19–21]. There was limited reporting of RA disease activity outcomes. For example, only three of the studies reported DAS28 scores [15, 20, 21] while four studies did not report on any type of disease activity assessment [16–19, 22]. Therefore, while the studies looked at patient beliefs, they did not analyse this in conjunction with typical measures of disease activity. There was a single study which was interventional in nature and included a qualitative analysis of the data, reporting that the majority of their patients were motivated to participate as they were looking for an alternative to conventional medicine and believed eating healthy could help them feel better.

### Relationship between common diets and RA symptoms

There were 55 studies identified which examined the impact of diets on RA symptoms, 14 interventional and 41 observational ( \* MERGEFORMAT Table 3). The studies were conducted globally including in Norway [23–25], United Kingdom [26, 27], Germany [28–31], Iraq [32], Finland [33–36], Sweden [37–41], Japan [42, 43], Malaysia [44], Italy [45, 46], Iran [47, 48] and Kuwait [49]. The majority of studies evaluated Mediterranean and fasting diets (i.e., intermittent or religious) with 19 and 13 studies identified, respectively. A lactovegetarian/ vegetarian diet was examined in nine studies and a vegan diet for eight. An elemental diet was investigated in only two studies and a western diet in three. Sample sizes varied from 10 to 413 participants. Dietary data collection methods included patient interviews, food questionnaires, food diaries and 24 h food recalls. The most common RA outcomes were DAS28, morning stiffness VAS pain, swollen joints, tender joints, ESR and CRP. However, some studies also observed health assessment questionnaires, physician global assessments and Ritchie’s articular index. Pain and DAS28 was the most highly reported outcome amongst all studies, closely followed by CRP, while morning stiffness was the least reported.

**Table 3:** Number of interventional and observational studies which reported an improvement in common rheumatoid arthritis outcomes for different types of diets

Type of diet	Number of studies	RA outcomes (number of studies which reported improvement/ number of studies which reported on outcome)						
		DAS-28	Morning stiffness	Pain	Tender joints	Swollen joints	CRP	ESR
Elemental [24, 26]	2	-^a^	0/1	0/1	2/2	1/2	0/2	0/2
Fasting [23, 25, 28, 30, 32, 41, 42, 44, 54, 55, 76–78]	13	5/6	3/5	5/9	5/7	5/7	0/5	2/4
Lactovegetarian/ Vegetarian [23, 25, 35, 39, 41, 77, 79–81]	9	1/3	1/3	2/5	1/2	1/2	0/2	0/2
Mediterranean [27, 28, 30, 38–40, 43, 45–53, 82–84]	19	10/16	2/3	3/8	0/2	0/2	3/9	3/9
Vegan [33–37, 39, 85, 86]	8	1/2	1/3	2/3	4/4	4/4	1/5	0/3
Western [29, 40, 87]	3	0/2	0/2	0/1	0/2	0/2	0/2	0/2

^a^The numerator displays the number of studies which reported a statistically significant improvement in a particular outcome while the denominator displays the number of studies which reported on that outcome. No score was given if there was no change or a decline in patient outcomes and a ‘-‘ denotes that the particular outcome was not measured

The Mediterranean diet was the most frequently studied compared with any other diet. There was a significant benefit in 10 of the 16 studies which measured DAS-28 score [28, 31, 40, 45, 49–53], two of the three studies which measured morning stiffness [27, 40], three of the eight studies which measured pain [27, 39, 49], three of the nine studies which measured CRP [31, 39, 40] and three of the nine studies which measured ESR [45, 49, 50]. In RA studies where patients fasted, five of the seven studies demonstrated clinical benefit in tender and swollen joints [25, 28, 32, 54, 55]. Fasting also positively impacted on disease activity according to DAS28, morning stiffness and VAS pain. Though RA activity and outcomes was observed in only a few vegan studies, there was a positive benefit on VAS pain, tender joints and swollen joints. The Western diet was the least studied regarding its impact on RA, and the studies which did explore this observed no improvement for any RA outcomes. CRP and ESR showed little to no improvement for any given diet.

### Relationship between observed dietary patterns and RA symptoms

The remaining studies did not observe patients in the setting of any specific diet or intervention but simply recorded food intakes to see whether particular food types or patterns of eating were associated with better RA disease outcomes. The results are summarised in (\* MERGEFORMAT Table 4). There were 12 studies in this group which were conducted in a variety of countries including Norway [56], Italy [57–59], United States of America [60], Finland [61], Japan [42, 43, 62, 63] and Turkey [64]. The oldest study was conducted in 1992 and the most recent in 2023. Sample sizes ranged from 10 to 441 participants where food frequency questionnaires were the most common form of data collection. However, studies also conducted patient interviews and asked participants to keep food diaries for review. The RA measurements of disease activity included DAS28, ESR, VAS for pain, duration of morning stiffness and a health assessment questionnaire. All studies were observational in nature except for two randomised clinical trials [58, 65] and two interventional studies; one where patients completed a diet of restricted caloric intake [42] and another where they were required to consume living foods such as fruits and vegetables [61]. The studies showed some positive impacts on RA symptoms for behaviours such as fish consumption at least twice per week, high consumption of unsaturated fats, lower caloric intake, and consumption of hypoallergenic foods. Almost all studies observed a positive clinical impact for the dietary pattern which they observed [42, 56–59, 61, 63–66], except for Hayashi et al., 2012 [62], who observed a negative impact for a diet with low fish and high vegetable fat consumption.

**Table 4:** Dietary patterns observed in studies and their effect on rheumatoid arthritis outcomes in patients

Studies	Dietary pattern observed	Effect on RA outcomes
Beyer et al., 2018 [56] Tedeschi et al., 2018 [66]	Consumption of fish at least twice per week	Positive impact
De Vito et al., 2023 [60]	Higher consumption of olive oil	Positive impact
Edefonti et al., 2020 [57]	High consumption of unsaturated fats	Positive impact
Hanninen et al., 2000 [61]	Consumption of only living foods such as raw fruits, vegetables, seeds and nuts	Positive impact
Hayashi et al., 2012 [62]	Low fish and high vegetable fat consumption	Negative impact
Iwashige et al., 2004 [42] Okumus et al., 2005 [64]	Reduction of caloric intake	Positive impact
Matsumoto et al., 2018 [43]	Lower fruit and higher monosaturated fatty acid consumption	Positive impact
Murakami et al., 2020 [63]	High consumption of fruits/vegetables and seafood	Positive impact
Sarzi-Puttini et al., 2000 [58] Van De Laar et al., 1992 [65]	Consumption of only hypoallergenic foods such as rice, cornmeal, hydrolysed milk, fresh pineapple and cooked apples	Positive impact

## Discussion

The most striking finding from this scoping review is that there is a paucity of research which examines the dietary beliefs of RA patients, even in the context of an intervention. Many studies are trying to establish what foods are best for symptom management, but very few are exploring patients’ beliefs which is a key aspect of behaviour change. Dietary beliefs play a key role in determining food choice and dietary patterns, especially when the individual’s experience of symptoms, either presence or absence, confirms the effects. Understanding the diet of RA patients could in turn lead to better patient education about anti-inflammatory foods and shape eating habits and ensure adherence to evidence based dietary interventions.

Diet has been explored as a possible cause of and method to manage disease symptoms in patients with other inflammatory conditions such as inflammatory bowel disease [67]. There has previously been a lack of conformity regarding what dietary approaches work best for these patients and it is only relatively recently that dietary guidelines have been released by the International Organization for the Study of Inflammatory Bowel Disease [68]. However, while there are established dietary guidelines for patients with inflammatory bowel disease, there is incomplete translation of this knowledge into best practice for patients. Studies examining dietary beliefs in patients with inflammatory bowel disease found that most patients and practitioners believed diet affected disease, yet only around half of the patients followed dietary advice provided by a clinician [69]. This demonstrates just how important it is to delve deeper into patient dietary beliefs and identify potential hurdles patient’s face when attempting to adapt to a particular diet. RA currently stands where inflammatory bowel disease was approximately 5 years ago, with a lack of evidence regarding patient beliefs and dietary guidelines. There is a need to continue research in this field, particularly as the number of new RA diagnoses shows no indication of slowing down [70]. The insight we can gain from the studies around inflammatory bowel disease also show us that without addressing patient beliefs there could be resistance to changing behaviour, even in the face of evidence based dietary guidelines.

This review identified that the use of validated instruments to explore dietary beliefs has been infrequent in existing literature. The lack of consensus regarding how dietary beliefs are measured has identified the need for a standardised methodology when it comes to patients with Inflammatory diseases like RA. A validated dietary beliefs questionnaire could make it easier to interpret data across studies It is also important to consider how other factors may impact dietary beliefs, such as gender, culture and ethnicity. In this review, there were no discernible differences between countries where studies were conducted as to whether patients held certain dietary beliefs. However, this likely reflects the very small number of studies. A deeper qualitative assessment of patients’ experiences of their diet and how they believe it affects their RA would now have high utility. Qualitative assessment could consider exploring enablers and barriers to adopting an anti-inflammatory diet or evidence-based diet in RA. By focusing on breaking down barriers which RA patients may have to adapting a beneficial diet, we can maximise patient adherence and reinforce positive habits for best clinical outcomes [10].

Despite limited evidence around dietary beliefs in RA, there is a considerable body of literature exploring the impact of diet on RA symptoms, particularly in the Mediterranean and fasting diets. These dietary patterns have been extensively explored in other conditions. Systematic reviews of a Mediterranean and fasting diet have shown beneficial outcomes for diseases such as cardiovascular disease, type two diabetes mellitus and stroke [71]. Fasting particularly has shown to reduce inflammation and promote longevity [72]. There has even been a recent systematic review for dietary interventions and RA where the findings are supportive of concepts emerging in this review, that dietary intervention may prove to be beneficial to patients [3]. Despite this emerging evidence base, there are still few studies to explore dietary beliefs. It is interesting, therefore, that there may be an evidence-implementation gap in modern rheumatological practice, and we are yet to establish what shape these interventions should take or how dietary advice may be personalised and adapted to patient’s gender, cultural or personal beliefs for maximal impact and sustainability. Furthermore, we see that patients do not adhere to recommended diets even where evidence is strong and come to their own conclusions about what foods work best in their own diets. Interestingly, although there is a body of evidence around the impact of diet on RA symptoms, in the limited studies on dietary beliefs identified, there was no mention of dietary patterns such as the Mediterranean diet. This loss of translation means that RA patients may be unaware of potential self-management options that or simply adopt an ad-hoc approach that may not best align with their beliefs, culture or dietary preferences. It is important that behavioural theories are explored to understand patient’s knowledge, attitudes, and beliefs about diet and how we can encourage them to intrinsically adapt positive lifestyle and dietary changes.

While the studies included in this review were conducted globally, there were no studies identified by the authors which looked into the dietary beliefs of Australian RA patients and only one study which assessed diet quality and RA outcomes in an Australian population [73]. This highlights a potential area of exploration for this research within an Australian cohort, especially given that the Australian population is multicultural, aging, relatively affluent with no food scarcity and has access to abundant and diverse food choices. Australian RA patients would, therefore, be in a unique position to reflect on their own dietary beliefs and perceptions about which types of foods and/or diets impact on their RA symptoms. It may also be beneficial to focus further on dietary beliefs in women, especially considering that women cook more than men on average and, therefore, their beliefs are likely to shape family food choices and eating habits [74].

There were several limitations of this scoping review. We only searched for English language articles, and this may have reduced our search results. When evaluating dietary beliefs, a variety of questions were asked across each of the studies, yet this review focused on the overarching themes and key beliefs for conciseness. Lastly, the different dietary patterns observed in the studies which did not meet clinical significance, were not covered by this review as they were too numerous. Despite these limitations, this review highlights that there is much literature which focuses on dietary interventions for patients with RA, with key diets such as Mediterranean and fasting being amongst the more researched for their benefits on RA activity. There was an overall lack of qualitative assessments of dietary beliefs in patients with RA. Further, while the studies explored RA outcomes, there was no uniformity in any single outcome which was explored by all studies. This review thoroughly explored the literature and has established a holistic picture of where RA and diet stands to date while also highlighting the knowledge gap around patient dietary beliefs related to their RA symptoms and disease. Subsequent studies that explore diet in patients with RA should be targeted at dietary beliefs. This groundwork can then develop into larger studies aimed at educating, expanding and effectively implementing dietary programmes for both patients and clinicians. Ultimately, this research may lead to patients having another potential avenue for disease management which they may be more likely to comply and with minimal risk of side effects.

## Conclusion

It is critical to understand patient beliefs regarding the impact of diet and dietary habits on their RA to help shape a dietary intervention which can be sustained by patients for best possible clinical impact. This scoping review identifies a significant gap in our understanding of patient’s beliefs about diet and RA disease activity both globally and in Australia. Future research can focus on understanding what obstacles patients face when presented with a potential anti-inflammatory diet, and how these obstacles can be reduced so that patients are more willing and able to comply with emerging evidence based nutritional interventions.

## Data Availability

Data sharing is not applicable to this article as no datasets were generated or analysed during the current study.
